# Plasma-Derived Fibronectin Stimulates Chondrogenic Differentiation of Human Subchondral Cortico-Spongious Progenitor Cells in Late-Stage Osteoarthritis

**DOI:** 10.3390/ijms160819477

**Published:** 2015-08-18

**Authors:** Chao Jiang, Pei Ma, Bupeng Ma, Zhihong Wu, Guixing Qiu, Xinlin Su, Zenan Xia, Zixing Ye, Yipeng Wang

**Affiliations:** Department of Orthopedic Surgery, Peking Union Medical College Hospital, Chinese Academy of Medical Sciences and Peking Union Medical College, Beijing 100730, China; E-Mails: jiangchao2189@sina.com (C.J.); dr_mapei@163.com (P.M.); mbp350579121@163.com (B.M.); wuzhihong139@139.com (Z.W.); qiugx@medmail.com.cn (G.Q.); suxinlin1988@163.com (X.S.); tienan523523@163.com (Z.X.); yezx06@mails.tsinghua.edu.cn (Z.Y.)

**Keywords:** plasma-derived fibronectin, cartilage regeneration, chondrogenesis, subchondral progenitor cells

## Abstract

Migration and chondrogenesis of human subchondral cortico-spongious progenitor cells (SPCs) are the key steps in the repair of microfracture-induced articular cartilage defects. The aim of this study was to evaluate the effect of human plasma-derived fibronectin (Fn) on the chondrogenic differentiation of SPCs, which was isolated from subchondrol cortico-spongious bone of late-stage osteoarthritis (OA) patients. SPCs were isolated and cultured for three passages. Stem cell surface antigens of SPCs were analyzed by flow cytometry. The osteogenic, chondrogenic and adipogenic differentiation potential were detected by histological staining. The chondrogenesis potential of SPCs with or without stimulation of either Fn or BMP-2 were studied by immunochemical staining and gene expression analysis. Cells isolated from subchondral bone presented to be positive for CD44, CD73, CD90, and CD166, and showed high capacity of osteogenic, adipogenic and chondrogenic differentiation, which suggested this cell population to be MSC-like cells. Stimulating with Fn increased the expression of SOX-9, aggrecan, collagen II while decreased the formation of collagen I by immunochemical staining. Gene expression analysis showed similar results. These results suggest that plasma-derived Fn can increase the chondrogenic differentiation of SPCs isolated from late-stage OA and improve cartilage repair after microfracture.

## 1. Introduction

Osteoarthritis (OA), known as chronic and progressive degenerative arthritis, is the leading cause of chronic disability in countries worldwide [1]. Although recent observations suggest OA to be a failure of the entire joint organ with the characterized pathological changes in the development and progression of OA (progressive cartilage loss, subchondral bone remodelling, formation of osteophyte and synovitis), articular cartilage degeneration and defect is still the main pathological change in OA [2]. Repairing the damaged cartilage is a good treatment option to ease joint pain, and regain the joint function, and a proper bridge between conservative symptomatic managements and the final joint replacement.

Mesenchymal stem cells (MSCs) are a group of cells that have the multiple differentiation potentials. By inducing MSCs’ chondrogenic differentiation under special conditions, cartilage repairing in OA seems to be promising [3,4,5,6,7,8]. These protocols generally call for three steps: cells harvesting *in vivo*, MSCs expanding *in vitro* and MSCs delivering to the lesion site, which would introduce related complications, possible disease transmission and cause high medical burden having to take multiple, complex steps [9]. Recently, minimally invasive microfracture is introduced as a kind of promising “one-step repair technique” which completes MSC harvesting and delivering in one single surgery. This technique was found to be simple, autogenous, and cost-effective for cartilage repair of OA patients, and therefore has attracted more and more attention [6,7,8]. However, there were also some issues about its application in patients with severe OA or in elders [10,11] due to markedly decreasing concentrations of MSCs in these patients [12,13]. Therefore, migration and chondrogenesis of MSCs from the subchondral area are the key to repair of cartilage defects in microfracture, and finding factors that recruit and promote chondrogenesis of MSCs is an important step in the clinical use of microfracture in OA cartilage defects. In 2013, Kulawig and colleagues reported that plasma-derived fibronectin (Fn) was a key factor in human serum to recruit MSCs and that it might be involved in subchondral MSC migration into cartilage defects after microfracture [6]. Although the chondrogenic stimulation capacity of plasma-derived Fn is still unknown, as an important composition of the extracelluar matrix (ECM), Fn is widely used in differential medium [14] with limited knowledge about its role as a scaffold for cell adhesion and differentiation, or in activating intracellular pathways for chondrogenesis. Based on all the publications above, we explored the potential role of plasma-derived Fn in stimulating differentiation of MSC from late stage OA knee into chondrocytes.

## 2. Result

### 2.1. Morphology and Cell-Surface Antigens of SPCs in Late Stage OA Knee

**Figure 1:**
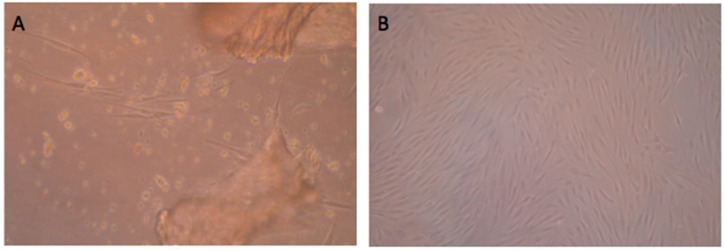
Morphology and cell-surface antigens of subchondral cortico-spongious progenitor cells (SPCs) in late-stage osteoarthritis (OA) patients. (**A**) Cells outgrew from subchondral bone chips and attached after 4‒6 days of culture (×400); (**B**) Cells (P_1_) at 90% confluence after 14 days of culture were spindle-shaped and fibroblast-like (×100).

**Figure 2:**
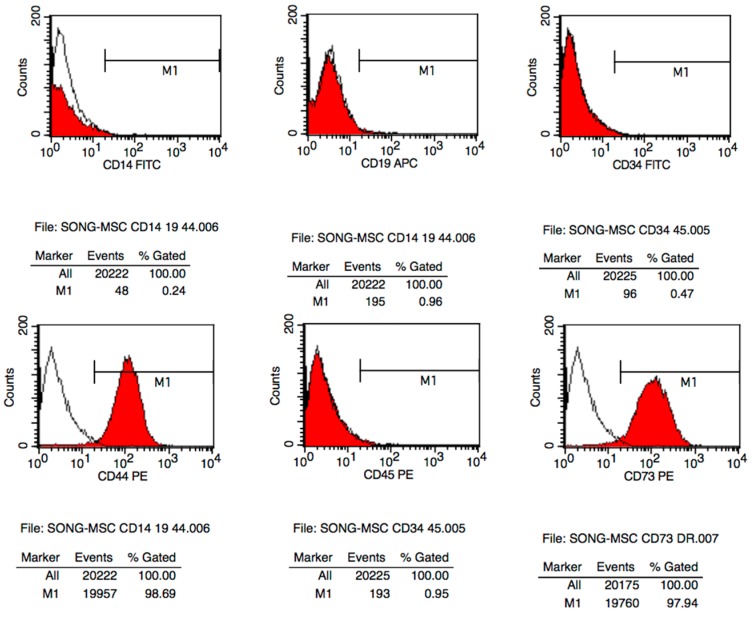

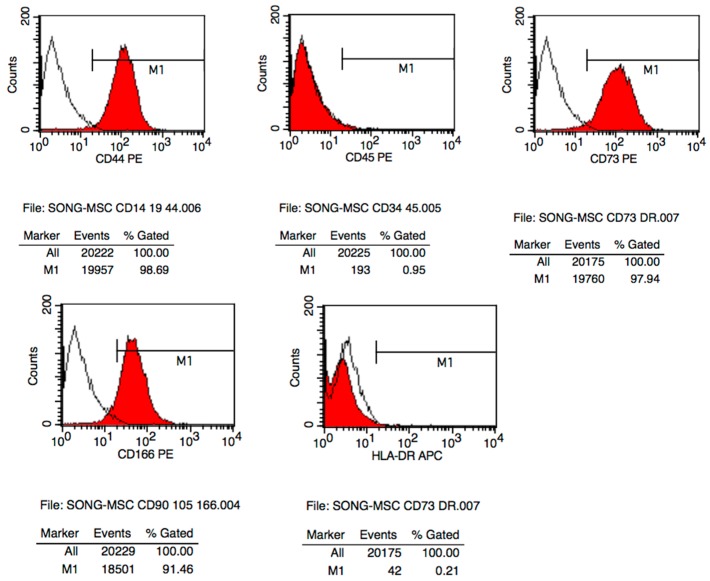
Cell-surface antigens of SPCs in late-stage OA patients. Cells were positive for CD44 (98.69%), CD73 (97.94%), CD90 (99.88%), CD166 (91.46%), partly positive for CD105 (6.13%) and CD146 (10.41%), and negative for CD14 (0.24%), CD19 (0.96%), CD34 (0.47%), CD45 (0.95%) and HLD-DR (0.21%).

Cells were grown out from subchondral bone chips and attached after 4‒6 days of culture, which were spindle-shaped and fibroblast-like (Figure 1A,B). They grew in monolayer, maintained a stable morphology with no signs of granulation. SPCs presented typical cell surface antigens known from mesenchymal stem cells. SPCs at passage 3 was homogenously positive for CD44 (98.69%), CD73 (97.94%), CD90 (99.88%), CD166 (91.46%). CD105 (6.13%) and CD146 (10.41%) were also detected, while the lipopolysaccharide receptor CD14 (0.24%), B-lymphocyte antigen CD19 (0.96%), haematopoietic surface antigen CD34 (0.47%), and the leukocyte common antigen CD45 (0.95%) and HLD-DR (0.21%) were not present (Figure 2).

### 2.2. Osteogenic or Adipogenic Differentiation Potential of SPCs

**Figure 3:**
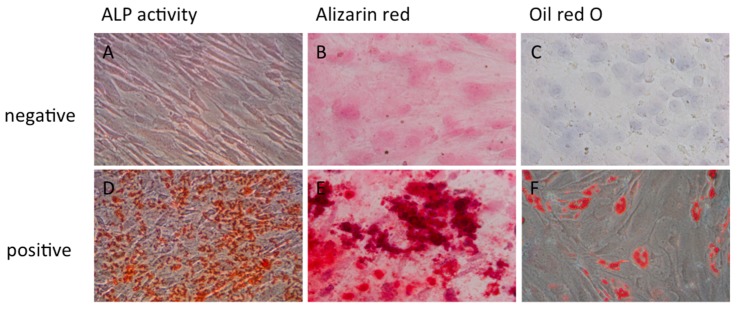
Histological analysis of SPCs undergoing osteogenic or adipogenic differentiation. By day 21, osteogenic induced SPCs cells showed alkaline phosphatase activity (**D**) and presence of mineralized matrix components (**E**); compared with non-differentiated cells (**A**,**B**); Adipogenic induced SPCs showed lipid droplets (**F**); while non-differentiated cells did not (**C**) (×400).

After 21 days of culture, cells stimulated with osteogenic inducing medium showed alkaline phosphatase activity, and evidence of mineralized extracellular matrix components by alizarin red staining, compared with non-stimulated cells (Figure 3A,B,D,E). Cells stimulated with adipogenic inducing medium showed lipid droplets by oil red O staining, while non-stimulated cells did not (Figure 3C,F).

### 2.3. Chondrogenic Differentiation of SPCs

**Figure 4:**
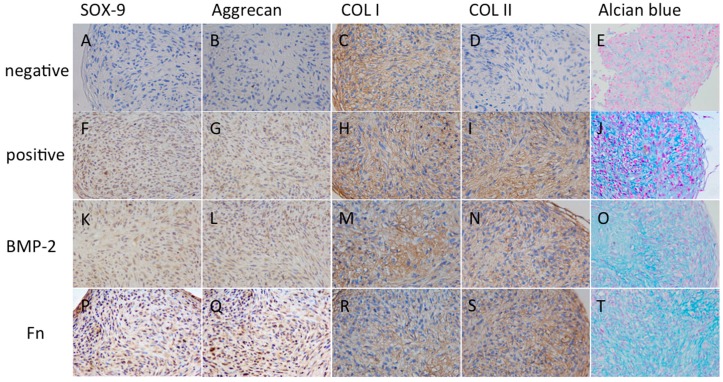
Histological and immunochemical analysis of SPCs undergoing chondrogenic differentiation with different stimulators. At day 28, SOX-9, Aggrecan, COL II and proteoglycans were evident in the positive group (**F**,**G**,**I**,**J**); BMP-2 group (**K**,**L**,**N**,**O**) and the Fn group (**P**,**Q**,**S**,**T**); negative group showed weak or absent staining (**A**,**B**,**D**,**E**); There was a strong staining of COL I in negative group (**C**); while a relatively weak staining in other stimulated groups (**H**,**M**,**R**). SOX-9, Aggrecan, COL II and COL I were brown immune-staining products with haematoxylin counterstain. Proteoglycans were stained blue with haematoxylin counterstain (×400).

After incubation for 28 days, histological analysis of micro-masses showed abundant proteoglycans in the positive control group (Figure 4J), while it was not shown in the negative control group (Figure 4E). The chondrogenesis-related genes COL II, aggrecan, SOX-9 and COL I in micro-mass cultures of MSC were detected by immunohistochemical staining. Only COL I was detected in MSC of negative control group (Figure 4C), which showed no existence of COL II, aggrecan or SOX-9 (Figure 4A,B,D), while they could all be detected in MSC of positive control group (Figure 4F–I).

### 2.4. The Effect of Fn on SPCs’ Chondrogenic Differentiation

To assess effects of Fn stimulation on chondrogenic differentiation, COL II, aggrecan, SOX-9 and COL I were also detected by immunohistochemical staining, together with proteoglycans staining by alcian blue in each group at day 28. In general, SPCs stimulated by either BMP-2 or Fn formed matrix rich in COL II and proteoglycan, and pellet which seemed more dense in Fn group. SPCs showed a decreased level of COL I (Figure 4M,R), and increased levels of COL II (Figure 4N,S), aggrecan (Figure 4L,Q) and Sox-9 (Figure 4K,P) in Fn and BMP-2 groups. SPCs stimulated by either BMP-2 or Fn showed a significantly increased staining of proteoglycans (Figure 4O,T).

### 2.5. Chondrogenic Differentiation Gene Expression of SPCs Stimulated by Fn

**Figure 5:**
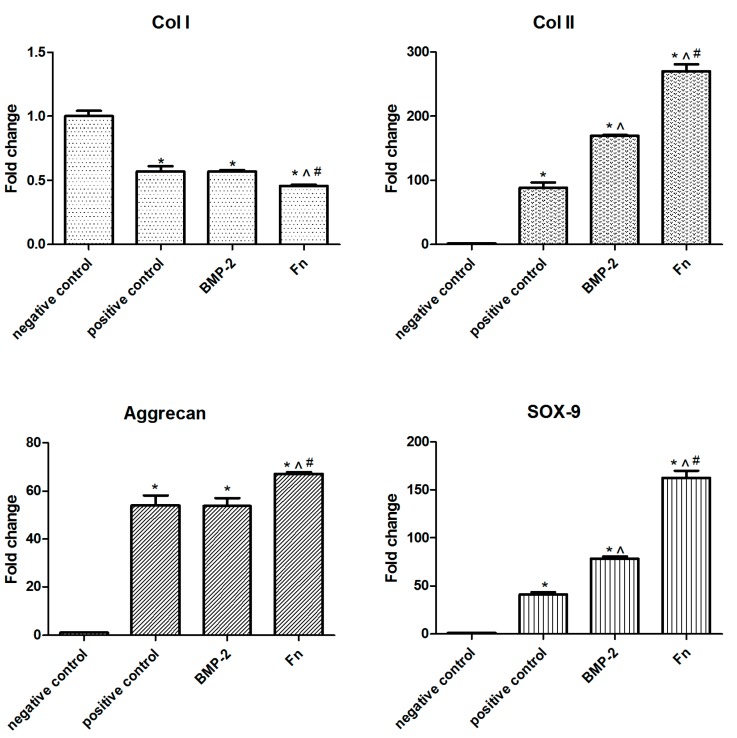
The gene expression of osteogenic marker gene COL I, as well as the chondrogenic marker genes COL II, Aggrecan, and SOX 9 stimulated by different chondrogenic factors. The mean of each triplicate well is plotted and the error bars represent SD. * compared to negative control group, *p* < 0.05; ^^^ compared to positive control group, *p* < 0.05; ^#^ compared to BMP-2 group, *p* < 0.05.

Genes related to chondrogenic differentiation were analyzed by RT-PCR at day 28. As for the fibrous tissue marker COL I, the mean expression level was 0.57 (positive control), 0.57 (BMP-2), and 0.46 (Fn), respectively (expression level in negative control group was designed as 1). As for the cartilage key marker gene COL II, the expression level was 88.11 (positive control), 168.97 (BMP-2), and 270.11 (Fn), respectively. The expression level of chondrogenic marker gene aggrecan was 54.04 (positive control), 53.76 (BMP-2), and 67.18 (Fn), respectively. The expression level of transcription factor Sox-9 was 41.29 (positive control), 78.09 (BMP-2), and 162.50 (Fn), respectively. Compared with negative group, all gene expression levels showed significant differences (*p* < 0.01). There were also significant differences between each stimulated groups (Figure 5). Treatment with BMP-2 or Fn stimulated chondrogenic differentiation of SPCs and the effect of Fn was stronger.

### 2.6. Proliferation and Migration of SPCs Stimulated by Fn

**Figure 6:**
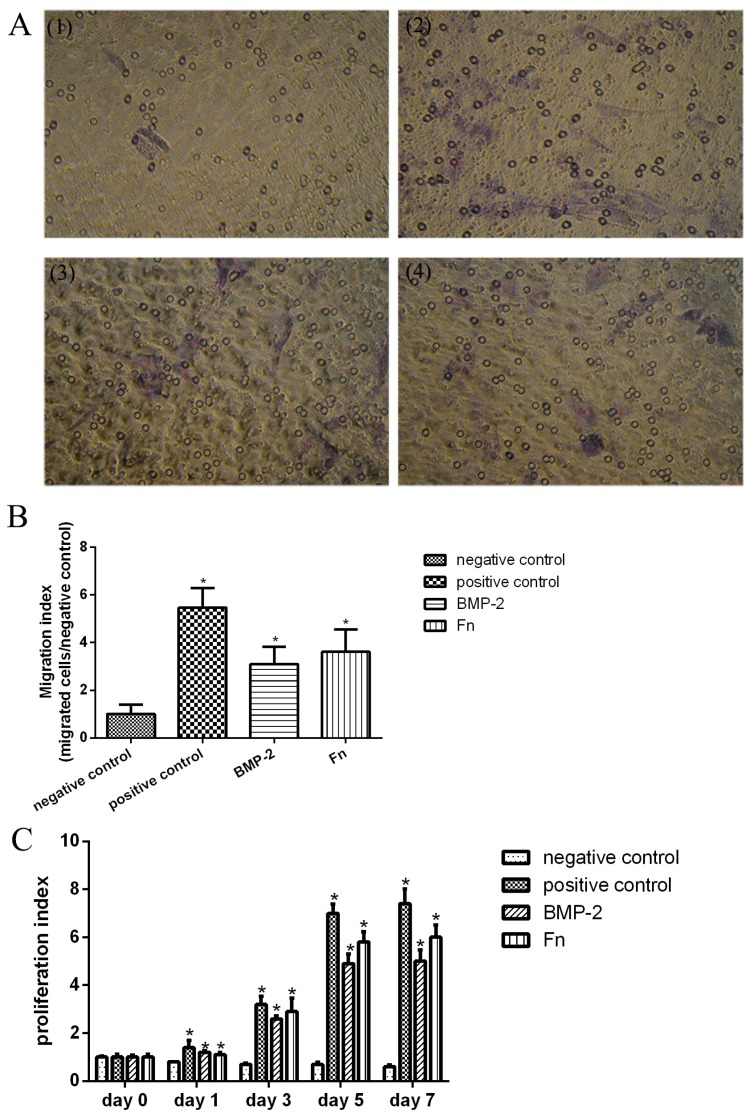
Quantification of proliferation and migration of SPCs cultured under different conditions. (**A**) SPCs migrated through the pores of the membrane and adhered at the bottom site of the polycarbonate membrane. Fenestra was the pore of the polycarbonate membrane, and SPCs were stained in purple and were spindle-shaped (×200). (**1**) Negative control; (**2**) positive control; (**3**) BMP-2 group; (**4**) Fn group; (**B**) The migration index in different groups. * Compared with negative control, *p* < 0.05; (**C**) Cell proliferation under different conditions. * Compared with day 0, *p* < 0.05. The mean of each triplicate well is plotted and the error bars represent SD.

Proliferation of SPCs had significantly increased since day 1 compared with day 0 in positive, BMP-2 and Fn groups (Figure 6C).

Fn could stimulate migration of SPCs significantly (Figure 6A). Few SPCs migrated through the pores of the polycarbonate membrane (Figure 6A(1)) and adhered at the bottom site of the membrane in negative control (migration index 1 ± 0.41). Stimulation with Fn significantly increased the migration index (3.6 ± 0.95, *p* < 0.05) (Figure 6A,B).

## 3. Discussion

Regeneration or repair of symptomatic articular cartilage defects has been at the forefront of regenerative medicine for decades [15]. A number of studies showed bone marrow MSCs improved symptoms of knee OA in terms of both short-term clinical outcomes and magnetic resonance observations of cartilage repair tissue scores, although the long-term clinical outcomes remained controversial [3,11].

In the current study, the human SPCs in late stage OA patients were confirmed to have the characteristics of MSCs by flow cytometry, displaying typical morphological features of MSCs as reported by previous studies [16], and representing the multipotent developmental capacity of MSCs to generate a diversity of mesodermal lineages, such as bone, fat, and cartilage. Using the micromass pellet culture system, SPCs from late stage OA knees possessed chondrogenic potential. Furthermore, this potential increased by adding different stimulators. The immunohistochemistry results showed the addition of plasma-derived Fn or BMP-2 increased the proteoglycans, COL II production compared with the positive control. Also, Fn group showed a larger increase, compared with the BMP-2 group. RT-PCR studies showed that SPCs treated with Fn or BMP-2 increased mRNA expression of COL II, aggrecan and SOX-9, again the increases of transcription were higher in the Fn group. Among these indexes, both COL II and aggrecan were major constituents of cartilage matrix, and SOX-9 played an essential role in cartilage formation. Fn stimulated the migration and proliferation of SPCs more clearly than negative control as well.

The highlights of the “one-step repair technique” are how to induce recruitment and/or migration, and chondrogenesis of progenitor cells, with chondrogenesis the most important step. Many different factors have been discussed as being responsible for this progression.

Bone morphogenetic proteins (BMPs)—members of the TGF-beta family—regulate cell proliferation, apoptosis, and differentiation [17]. They have been shown to play a crucial role in chondrogenic development of mesenchymal progenitors [16], stimulating the synthesis of cartilage matrix components by articular chondrocytes [18]. Here, our results were in line with these conclusions. Over the last few decades, Fn has been proven as a key factor in human serum to enhance mesenchymal progenitor cell migration and proliferation among a wide range of cell types and tissues [6,19], including subchondral mesenchymal progenitor cells from the femur head of healthy donors post-mortem [20]. As a part of the matrix of various tissues, Fn has frequently been a topic of extracellular matrix research. It has shown significant migratory and proliferative effects on subchondral progenitor cells which relied on both chemotactic and haptotactic activity [20], and therefore promotes the formation of cartilage-like repair tissue containing collagen type II [14,19]. Our results were in line with the previous conclusions. In this study, SPCs from subchondral bone of OA donors also showed increased migration and proliferation capacities by stimulation of Fn. Regarding differentiation, some previous studies observed inhibitory effects of plasma-derived Fn on chondrogenic differentiation of chicken limb bud mesenchymal cells [21], while others reported plasma-derived Fn had no potential effect on chondrogenic differentiation [20]. However, we could show that Fn is associated with chondrogenic differentiation of SPCs from the subchondral bone. The difference in the differentiation profile of the progenitor cells could be induced by several reasons. Firstly, MSCs from OA donors showed reduced proliferative capacity and chondrogenic and adipogenic differentiation, compared to those in the normal population, which was not age- or site-dependent, but was associated with disease [22]. SPCs here were isolated from the subchondral bone of OA patients, while subchondral mesenchymal progenitor cells from the femur head of healthy donors post-mortem were examined in a previous study [20]. Secondly, the concentration of Fn used here was different from the previous study (25 *vs*. 15 μg/mL) [20]. There was lack of knowledge regarding effects of treatment with higher doses of Fn, which needed further study Thirdly, these could also be partially deduced to various isoforms of Fn, which resulted in the determination of impact by the selected type of molecule and the proper concentration. Although highly speculative, one possible reason is that Fn plays a structural role by providing a scaffold for cell adhesion and differentiation. Another possible reason is that Fn signals through integrins to activate intracellular pathways that regulate changes in gene expression during chondrogenesis [23,24], which was also reflected by our mRNA results. A third probable reason was that Fn was involved in the organization of the cartilage matrix. The Fn matrix was shown to support deposition of collagens, latent TGF-β binding proteins and other ECM proteins [14]. Our results showed the increasing level of collagen II and aggrecan, which was in line with previous studies. Therefore, Fn could be a potential application for the histological outcomes of cartilage regeneration after using the “one-step repair technique”. Besides, from data of gene expression analysis, we found that plasma-derived Fn is not inferior to BMP-2 in chondrogenic inducing capacity. However, the mechanisms of Fn mediated stimulation of chondrogenesis need further study, which is a limitation of this study.

In conclusion, this study confirms for the first time that plasma-derived Fn has chondrogenic differentiation impact on human SPCs, and is a potential source of autologous growth factors that is clinically applied using the “one-step repair technique” in areas such as microfracture for the treatment of focal articular cartilage defects in patients with OA.

## 4. Experimental Section

### 4.1. Isolation and Cultivation of SPCs

Human subchondral bone chips with 3‒5 mm diameters were obtained from the tibial platform of 13 donors (8 females, 5 males; age 54‒79 years, average age 66.8 years) undertaking total knee arthroplasty due to late stage OA. The study was approved by the ethics committee of Peking Union Medical College Hospital. All patients who had late stage OA and underwent total knee arthroplasty provided written informed consent. MSC was then isolated and cultured as described previously [25]. In brief, subchondral bone chips were washed with phosphate buffered saline (PBS), placed in cell culture flasks (Costar, Corning Scientific, New York, NY, USA), and cultured in DMEM (Hyclone, Logan, UT, USA) containing 10% fetal bovine serum, 100 U/mL penicillin, 100 μg/mL streptomycin, 2 mM l-glutamine (all Invitrogen, San Diego, CA, USA) at 37 °C. After 4‒6 days of incubation without medium change, spindle-like and adherent cells began to come up around bone chips. The media was then exchanged for the first time, and later exchanged every 2‒3 days. At 80%‒90% confluence, cells were harvested using trypsine/EDTA (Invitrogen, Karlsruhe, Germany) and re-plated at a density of 6000 cells/cm^2^.

### 4.2. Flow Cytometric Analysis

Cells (250,000 cells, passage 3) was harvested and washed by PBS/0.5% bovine serum albumin (BSA) twice, then incubated with phycoerythrine (PE), fluorescein isothiocyanate (FITC) or allophycocaine (APC)-labeled monoclonal mouse anti-human antibodies CD14 FITC, CD19 APC, CD34 FITC, CD44 PE, CD45 PE, CD73 PE, CD90 APC, CD105 FITC, CD146 PE, CD166 PE, HLA-DR APC (all Abcam, Cambridge, UK) for 15 min on ice. Staining of cell surface antigens was analyzed using FACS Calibur (Becton and Dickinson). Apoptotic cells were excluded from analysis using propidium iodide (PI). Unstained cells served as negative, and CD 34-PE/FITC-stained cells served as isotypic control.

### 4.3. Osteogenic and Adipogenic Differentiation of SPCs

To induce osteogenic and adipogenic differentiation, SPCs (80,000 cells, passage 3) were cultured in a 24-well plate with either osteogenic (CCM007 + CCM008; R&D Systems, Inc., USA) or adipogenic (CCM007 + CCM011; R&D Systems, Minneapolis, MN, USA) differentiation medium. Cells were cultured for 21 days and medium was changed every three days.

### 4.4. Chondrogenic Differentiation of SPCs

Chondrogenic differentiation of SPCs (250,000, passage 3) was performed in a 96-well U-bottom plate by high-density pellet cultures as previously described [26]. Cells were incubated in different culture as follows: basic media (CCM005; R&D Systems, Inc., USA) without chondrogenic differentiation supplement (negative control group), chondrogenic differentiation media (CCM005 + CCM006; R&D Systems, Inc., USA) (positive control group), chondrogenic differentiation media with 100 ng/mL BMP-2 (R&D Systems, Inc., USA) (BMP-2 group), chondrogenic differentiation media with 25 μg/mL human fibronectin (Sigma, Saint Louis, MO, USA) (Fn group). Chondrogenic differentiation media was basic media with chondrogenic differentiation supplement, which was used without adding additional serum. The medium was exchanged every three days and cells were maintained for up to 28 days.

### 4.5. Histology and Immunohistochemistry

Osteogenic differentiation (*n* = 9) was assessed by Alizarin red staining of mineralized matrix components and alkaline phosphatase activity (ALP kit, Nanjin-Jiancheng, Nanjin, China). The lipid droplets were stained by Oil red O (Sigma, Saint Louis, MO, USA) to assess the adipogenic differentiation (*n* = 9).

The cryo-sections (6 μm; *n* = 9) were prepared for assessing chondrogenic differentiation and stained by Alcian Blue (Sigma). Besides, the cryo-sections were fixed and incubated with the primary mouse anti-human type I collagen (COL I) antibody, type II (COL II) collagen antibody, and aggrecan (Agg) antibody (all Abcam, Cambridge, UK) for 40 min. Mouse IgG served as controls. Subsequently, cells were colormetrically detected with 3-amino-9-ethylcarbazole (EnVisionTM; Dako, Glostrup, Denmark) according to the manufacturer’s instructions, followed by counterstaining with hematoxylin (Merck, Darmstadt, Germany).

### 4.6. Quantitative RT-RCR

Total RNA (12 pellets per experimental condition) was isolated with Trizol reagent (Invitrogen), and 2 μg RNA was reversely transcribed with cDNA Synthesis Kit (TaKaRa, Dalian, China) according to the manufacturer’s recommendations. The relative expression level of the housekeeping gene glyceraldehyde-3-phosphate dehydrogenase (GAPDH) was used to normalize the samples. RT-PCR was performed with 1 μL of each cDNA sample in triplicates using 7500 Real-Time PCR System (Applied Biosystems) with SYBR green PCR Core Kit (Applied Biosystems, Foster City, CA, USA). Relative quantitation of the marker genes (Table 1) was performed according to the ^ΔΔ^*C*_t_ method and is given as fold change compared to controls.

**Table 1:** Sequences of the primers used in RT-PCR.

Gene	Forward Prime	Reverse Primer	Tm (°C)	Product Size (bp)
*Collagen 1a1*	CGATGGCTGCACGAGTCACAC	CAGGTTGGGATGGAGGGAGTTTAC	62	180
*Collagen 2a1*	CCGGGCAGAGGGCAATAGCAGGTT	CAATGATGGGGAGGCGTGAG	58	128
*Aggrecan*	CCCAAGAATCAAGTGGAGCCG	ACACGATGCCTTTCACCACGA	64	254
*SOX-9*	AGCGAACGCACATCAAGAC	CTGTAGGCGATCTGTTGGGG	58	85
*GAPDH*	ATTTGGTCGTATTGGGCG	TGGAAGATGGTGATGGGATT	57	204

### 4.7. Proliferation and Migration Assay

For cell proliferation assay, SPCs were seeded (2000 cell pre well) in 96-well plate and incubated for 16 h. Then, attached cells were washed twice with PBS, and incubated under different conditions, *i.e.*, MSC medium without FBS (negative control), MSC medium with 10% FBS (positive control), 100 ng/mL BMP-2 in MSC medium containing 1% FBS, 25 μg/mL human fibronectin in MSC medium containing 1% FBS. At time point of 0, 1, 3, 5, 7 days, 10 μL CCK8 was added to the medium and incubated for an additional 2 h. The optical density at a wavelength of 450 nm was analyzed with a microliter plate reader.

For cell migration assay, SPCs were starved in basal medium for 12 h, and 2 × 10^4^ SPCs were suspended in 400 μL medium with 1% FBS and placed in the upper chamber. Five hundred microliters of medium used in different groups (the same with cell proliferation assay) was placed in the lower compartment. After 6 h incubation at 37 °C, the lower side of the filter was washed with PBS and fixed with methol. For quantification, cells were stained with Giemsa. Cells migrating into the lower chamber were counted manually in 10 random microscopic fields (×200).

### 4.8. Statistical Analysis

The Kolmogorove-Smirnov method was applied for testing normal distribution of the data. The *t*-test was applied for normally distributed data, while the Mann-Whitney *U*-test was used for data that failed normality testing. In all tests, *p* values less than 0.05 were considered statistically significant.
